# Synaptic Plasticity in Neurodegenerative Diseases: Impact of Exercise as Promising Therapeutic Tool

**DOI:** 10.3390/cells15020197

**Published:** 2026-01-20

**Authors:** Gabriele Farina, Gianmarco Fenili, Maria Paola Paronetto, Clara Crescioli

**Affiliations:** 1 Department of Movement, Human and Health Sciences, University of Rome “Foro Italico”, Piazza L. de Bosis 6, 00135 Rome, Italy; g.farina2@studenti.uniroma4.it (G.F.); g.fenili1@studenti.uniroma4.it (G.F.); mariapaola.paronetto@uniroma4.it (M.P.P.); 2 Laboratory of Molecular and Cellular Neurobiology, Fondazione Santa Lucia IRCCS, Via del Fosso di Fiorano, 64, 00143 Rome, Italy

**Keywords:** synaptic plasticity, exercise, neurodegenerative diseases

## Abstract

**Highlights:**

**What are the main findings?**

- Synaptic plasticity dysfunction is the basis for neuroimmune inflammation/neurodegeneration.
- Exercise can counteract synaptic plasticity decline/neuroinflammation and maintain neuroimmune homeostasis.

**What are the implication of the main findings?**

- Exercise should be included as a promising intervention for supporting and preserving neuroplasticity

**Abstract:**

Neurodegenerative diseases are distinguished by synaptic dysfunction and chronic neuroinflammation, which accelerate neuronal loss and impair network resilience. Synaptic plasticity, that is, the ability to adapt to changes, is progressively lost. This ability is part of hormesis, an adaptive biphasic response, nowadays acknowledged as a promising tool in chronic degenerative diseases, since it offers a framework for personalized interventions. Growing evidence supports exercise as a powerful approach for managing neurodegenerative disorders, due to its capacity to enhance neuroplasticity through the direct targeting of the biomolecular processes involved. Indeed, regular exercise can drive many molecular mediators and signals toward neuroplasticity improvement, potentially slowing neurodegeneration. This narrative review focuses on exercise as a promising therapeutic approach in neurodegenerative diseases, based on its ability to shape synaptic plasticity at the molecular level. Some biomediators involved in synaptic plasticity function/dysfunction and neuroinflammation/neurodegeneration are addressed as therapeutic targets of exercise, and different exercise regimens are discussed as specific therapeutic interventions to contain the burden of some neurodegenerative conditions. Some clinical trials including exercise in the treatment of neurodegenerative diseases are summarized. Since no definitive disease-modifying cure exists for these illnesses, exercise’s ability to shape synaptic plasticity emerges as a highly attractive therapeutic approach.

## 1. Introduction

Neurodegeneration represents a growing cause of mortality and morbidity in the general population, especially in the elderly, imposing substantial clinical and public health burdens [1]. Although neurodegenerative diseases exhibit different courses, symptoms, and features, they share a common ground related with progressive decline in neuronal functionalities, due to genetic mutations, protein dysfunction and/or environmental factors [1]. The progressive degeneration of neurons in the central nervous system (CNS) significantly affects the quality of life (QoL) of patients and caregivers, highly impacting healthcare systems.

A main and common trait of neurodegenerative diseases (either primary or secondary to other pathologies, i.e., type 2 diabetes, ischemic stroke, or other neurovascular accidents) is the loss of synaptic plasticity. Synapses can adapt to external changes through two main mechanisms: long-term potentiation (LTP) and long-term depression (LTD). These mechanisms involve the strengthening or weakening of synaptic connections, respectively, thereby regulating neurotransmitter release and post-synaptic receptors. [2].

An intact synaptic plasticity plays an important role at the interface between motor and cognitive systems within brain network dynamics, and integrates multiple neural systems to warrant neuroprotection [3]. Synaptic plasticity dysfunction seems to precede the loss of adaptive cognition/movement coordination and skill acquisition in aging and neurodegenerative diseases, i.e., Alzheimer’s disease (AD), Parkinson’s disease (PD), Huntington’s disease (HD), and amyotrophic lateral sclerosis (ALS), representing, therefore, a critical target for early intervention [4].

Any intervention able to interfere with transcription and molecular factors essentially involved in synaptic plasticity and neuroprotection, i.e., nuclear factor kB (NF-kB), cyclic adenosine monophosphate (cAMP)-response element-binding (CREB) protein, nuclear factor erythroid 2-related factor 2 (Nrf2), the neurotrophin brain-derived neurotrophic factor (BDNF), Ca^2+^ signaling, heat shock proteins, antioxidant antiapoptotic proteins, and synaptic scaffolding proteins [5], retains potential as therapeutic intervention.

Regular exercising, both aerobic and resistance training, is capturing growing interest as a non-pharmacological therapeutic strategy since it can enhance synaptic plasticity by modulating neurotrophic, metabolic, and anti-inflammatory mechanisms [6]. The first evidence pertaining to exercise as a tool to potentiate LTP and decrease LTD dates to about twenty years ago [7]. From that point on, many studies expanded these findings to identify other molecular mechanisms as exercise-mediated targets. This narrative review aims to discuss exercise as a novel tool in the therapeutic approach to neurodegenerative diseases due to its ability to improve synaptic plasticity at molecular level. To this purpose, the function/dysfunction of synaptic plasticity is first reviewed in the context of neurodegeneration and the neuroinflammatory environment. Then, different exercise regimens are discussed as specific therapeutic interventions to contain the burden of some neurodegenerative diseases. Furthermore, an up-to-date summary of clinical trials including exercise in the therapeutic approach of neurodegenerative diseases is provided.

## 2. Synaptic Plasticity and Hormesis

Synaptic plasticity is classically acknowledged as responsible for thought–action coupling in a bidirectional mode, where cognition shapes motor behavior, and motor experience fine-tunes cognitive representations [2]. In other words, synaptic plasticity represents the neural basis of adaptive behavior and, therefore, it has been classically associated with learning and memory. Adaptive behavior is a complex process that involves modifications and adjustment within the individual neurons in many brain regions to allow specific functions. As examples, in the motor cortex, synaptic plasticity allows the fine-tuning of movement pattern through practice; in the basal ganglia and cerebellum, it shapes plastic changes associated with cognitive factors, like motivation or attention; and in the prefrontal motor circuits, synaptic plasticity regulates cognitive control over movements [8]. Therefore, acquiring a particular adaptive behavior toward a goal-directed purpose is a very complex process, at the basis of learning and memory, involving cognitive flexibility and adaptive neural circuit remodeling [2,3].

The adaptive response of neural cells is a part of a biological process, common across plants and humans, known as hormesis, which consists of a biphasic adaptive dose–response to a wide range of stimuli, usually with a low dose challenge improving general health [9]. The concept of hormesis has been historically debated. Nowadays, the scientific community acknowledges it as a promising tool in different research areas, and particularly in the context of chronic degenerative diseases [10,11,12,13,14]. Indeed, the principle of hormesis offers a framework for personalized interventions in the therapeutic approach to neurodegenerative conditions and cognitive decline [15].

It is worth recalling that neurodegeneration is tightly linked with neuroinflammation. Inflammation of the CNS is a physiological reaction of microglia, the CNS’ innate immune cells, in defense to harmful stimuli like traumas or infections. If microglia overactivation is incorrectly protracted, likely because of persistent stimuli (i.e., endogenous/environmental factors or genetic susceptibility), this induces synaptic alteration, along with aberrant signaling cascades, the alteration of brain–blood barrier (BBB) permeability, and a huge release of reactive oxygen species (ROS), nitrogen species, cytokines, and chemokine [16,17,18,19]. Thus, the protracted overactivation of the CNS’ resident immune cells causes neurotoxicity associated with mitochondrial dysfunction, synaptic loss, and neuronal death; furthermore, the clearance failure of misfolded proteins and the aberrant protein accumulation, such as amyloid beta (Aβ) in AD or α-synuclein aggregates in PD, and enhances microglia chronic activation and inflammation [18,20,21,22]. Thus far, neuroimmune/inflammatory processes appear to happen earlier than protein aggregation.

In this scenario, it is not surprising that any of the therapeutic approaches aimed to target protein aggregates and plaque formation presents a high rate of unsuccess and fail to counteract disease progression [18]. Remarkably, interventions aimed to target early inflammatory cascades, that, in turn, unbalance excitatory and inhibitory synaptic transmission, represent a promising approach in neurodegenerative pathogenesis [19,23]. As general examples, the interaction between the inflammasome (NLRP) pathway, a cytosolic multiprotein complex, and toll-like receptor (TLR) system, one of the main triggers of inflammation, plays a pivotal role in increasing proinflammatory cytokines and oxidative stress molecules in response to pathogen-associated molecular patterns (PAMPs) or damage-associated molecular patterns (DAMPs) [18,24,25]. In this context, given the role of NLRP3 in PD, inhibiting NLRP3 signaling, i.e., with specific inhibitors (microRNA, MCC950, and inzomelid), could be a therapeutic strategy, as shown in animals and humans [26,27,28].

The increase in oxidative stress due to reduced antioxidant protein activity and defects in mitochondrial respiratory chain complex activity are among the main causes described in neurodegenerative diseases such as PD, AD, and HD [29,30]. Some neurotrophic cascades, i.e., BDNF- or nerve growth factor (NGF)-dependent ones, are so critical to maintaining the integrity of synaptic plasticity that they have been experimentally studied as therapy to upregulate neurotrophic signaling against neurodegeneration [31]. In particular, the glial cell line-derived neurotrophic factor (GDNF) showed neuroprotective effects when implanted in an experimental model and in humans affected by PD or HD [18]. Another critical signaling is associated with the NF-kB, a transcription factor associated with synaptic plasticity [32] and tightly connected with resident cells’ overactivation during neurodegenerative pathogenesis [18].

In this scenario, among other hormetic interventions, like intermittent fasting, cognitive stimulation, dietary phytochemicals, and exercise could be considered a promising tool to counteract neurodegeneration, targeting several biomolecular paths.

## 3. Exercise Regimens: Some Examples

Exercise is characterized by planned, structured, and repetitive physical movements aimed at improving or maintaining various components of physical fitness [33,34]. Exercise has emerged as key modulator of brain plasticity, providing a promising strategy for mitigating cognitive decline and related disease [35]. It ameliorates neuronal activity and connectivity, promoting brain plasticity by the facilitation of information transfer and the modulation of neural networks [36], and enhances cognitive performance, learning [37], and memory [38]. Of note, the effects of exercise, i.e., in preventing or treating disease, is influenced by factors such as the type, duration, frequency, and intensity of the exercise [37,39]. Designing evidence-based exercise prescriptions for neurodegenerative disease prevention requires balancing efficacy with adherence and safety considerations [40,41,42]. The frequency, intensity, time, type, volume, and progression (FITT-VP) model provides a useful structure for exercise prescription development [41].

There are four important categories of exercise: aerobic, anaerobic, resistance, and mind–body (or neuromuscular) exercise [42].

### 3.1. Aerobic Exercise

The first refers to the type of repetitive, structured physical activity that requires the body’s metabolic system to use oxygen to produce energy [43]. It improves the capacity of the cardiovascular system to uptake and transport oxygen. Aerobic activity can be undertaken in many different forms, such as running, cycling, and swimming, and has been demonstrated to elevate heart rate and enhance cerebral blood flow, facilitating the delivery of vital nutrients and oxygen to the brain [44].

In healthy adult men, acute aerobic exercise increases serum BDNF [45]. Interestingly, vigorous-intensity (80% heart rate reserve, HRR), long-duration (40 min) exercise offers the greatest probability of a significant BDNF increase compared to other exercise intensity/duration modalities [45]. Older subjects engaged in both home and in-person based training (4 months, 150 min/week, 12–14 Rate of Perceived Exertion, or RPE, on Borg’s 6–20 scale) show a significant increase in cerebral blood flow and greater connectivity in the hippocampus vs. control [46].

Acute aerobic exercise sessions (70–80% heart rate or HRmax, 30 min) increases BDNF concentration in young adults. To date, serum BDNF seems to depend on different levels of physical activity [47]. Aerobic exercise on treadmills (6 months, 40 min, 60–80% HRmax) in elderly subjects seems more appropriate for downregulating proinflammatory cytokines like IL-6 and TNFα compared to resistance training [48].

Treadmill exercise and running (7 days/week, 6 weeks, 30 min, 2 m/min load increased by time) delayed cognitive decline by neurogenesis’ increase and BDNF expression upregulation in the hippocampal dentate gyrus, in a rat vascular dementia model [49]. This effect was greater in adult rats compared to young rats. Moreover, TrkB expression in the hippocampal dentate gyrus was higher in adult rats; vice versa, neurogenesis in young rats increased more compared to adult-age rats [49]. Aerobic exercise (1 month, continuous and self-regulated exercise) in mice is sufficient to increase BDNF expression in the hippocampus, both at the mRNA and protein level, likely due to beta-hydroxybutyrate (BHB). This ketone body acts as an epigenetic modulator and endogenous mediator, increasing histone acetylation and, thereby, permitting BDNF transcription [50]. In general, besides the hippocampus, which is the most studied, the brain areas reported to express increased BDNF after exercise are involved in learning, memory, mood, and motor control, like the prefrontal cortex, motor cortex, striatum, and cerebellum [44].

Studies on neurotrophin (NT)-4/5, closely related to the homodimeric polypeptide growth factors family [51] are reported only in animal models, with some discrepancies. In adult male rats this molecule was upregulated by a treadmill protocol (5 days/week, 4 weeks not defined intensity), whereas in mice no significant effect was observed with a protocol of the same duration and frequency [52]. An aerobic protocol (4 weeks, 5 days/week, 1 h/day 10 m/min load) upregulated the expression of IGF-1 and activated the IGF-1 receptor(1R)-phosphatidylinositol 3-kinase (PI3K)/protein kinase B (Akt) signaling pathway in the skeletal muscle of infarcted mice [53]. IIGF1R-mediated signaling modulated important neurophysiological aspects in the CNS, including neurogenesis, synaptic plasticity, and complex cognitive functions [54]. Of note, several lines of evidence have demonstrated that IGF1R-mediated actions against various neurotoxic cues involve the PI3K/Akt and mitogen-activated protein kinase (MAPK)/ERK1/2 signaling path [55]. Along with IGF-1, VEGF plays a key role in promoting angiogenesis and hippocampal neurogenesis [56,57]. VEGF derived from skeletal muscle has the potential to induce direct effects on nuclear pore complex (NPCs) or influence downstream pathways that affect neurogenesis [58]. Aerobic exercise on treadmills (2 weeks, frequency and intensity not defined) increased VEGF levels in the hippocampus and this was associated with neurogenesis, improved hippocampal blood flow, better running performance (speed and endurance), and a protective response to hypoxia [59]. Since VEGF can cross the BBB, it is possible that circulating VEGF asserts its effects directly on NPC, inducing cell proliferation or increasing cell survival [60].

### 3.2. Anaerobic Exercise

Anaerobic exercise has been defined by the American College of Sport Medicine (ACSM) as intense physical activity of very short duration, fueled by the energy sources within the contracting muscles and independent of the use of inhaled oxygen as an energy source. Without the use of oxygen, cells revert ATP formation via glycolysis and fermentation. This process produces significantly less ATP than its aerobic counterpart and leads to the build-up of lactic acid. The sirtuin (SIRT)1/PGC-1α/FNDC5/BDNF pathway is targeted by exercise through lactate, improving learning and memory [61,62]. Moreover, lactate contributes to exercise-induced brain resilience through a distinct mechanism involving enhanced synaptic protein expression and the modulation of gene networks associated with synaptic plasticity and axonogenesis [62]. Recent studies have confirmed the potential link between exercise-induced metabolic factors (such as β-hydroxybutyrate and lactate) and muscle-derived myokines (such as glycosylphosphatidylinositol-specific phospholipase D1 or Gpld1 and irisin) with improvements in brain function [62]. The anaerobic exercise regimen typically involves fast twitch muscle and includes activities such as sprinting, high-intensity interval training (HIIT), and power lifting [63]. Anaerobic exercise (trained for 6.8 ± 3.1 years, other training parameters not defined) in adult sprinter athletes show higher gray matter volume in the region of the basal ganglia, including the right caudate, the left claustrum, striatum, and thalamus, suggesting effects on brain plasticity [64]. Sprint training (2 weeks, 3-session/week, 42 min duration, intensity not defined) increased BDNF levels and working memory in young females [65].

HIIT exercise (7 weeks, 5 days/week, ten cycles of 3 min/85% maximal speed followed by 2 min at 45% of maximal speed) in mice has positive effects on brain functions, through the enhancement of VEGF, the angiogenesis signaling pathway (p/t-AKT/ENOS/VEGF), the mitochondrial biomarker (SDHA), and the metabolic protein (p/t-CREB, p/t-HSL and lactate dehydrogenase or LDH) in the hippocampus [66]. In healthy rats, anaerobic training (8 weeks, 5 days/week, 28 min duration, running speed fixed 80–100% of the maximal speed) seems better than aerobic training in terms of increasing brain plasticity markers (FNDC5, VEGF, PGC1- 1α, and TrkB) in the hippocampus, without improving cognitive functions [67].

### 3.3. Resistance Exercise

Resistance exercise, also known as strength exercise, refers to specialized physical conditioning methods that apply a variety of resistance loads, different movement speeds, and various training modes, including weight machines, free weights (barbells and dumbbells), elastic bands, medicine balls, and plyometrics [68]. It consists of activities that enhance both muscle strength and endurance, and has a substantial impact in terms of increasing neuroplasticity, potentially through mechanisms distinct from those of aerobic exercise [69].

In healthy untrained adult males, 5 weeks of resistance training elicited a transient but robust increase in circulating BDNF. The acute BDNF response was significantly amplified after training, with post-exercise levels rising to 77% from 32% at the baseline, and with a faster return to resting concentrations during recovery; the magnitude of the BDNF rose immediately after exercise [70]. Another study on the role of moderate- and high-intensity acute resistance training in young adult males shows significant elevations in circulating IGF-1 compared with a non-exercising control. However, these increased IGF-1 levels declined rapidly (20 min) after exercise and no significant association was found between IGF-1 change and improvements in cognitive or electrophysiological performance [71]. This study suggests that these improvements are due to additional mechanisms, such as increases in central BDNF, improved synaptic excitability, enhanced blood flow, and local IGF-1 signaling within the brain. Both land and water resistance training (12 weeks, three times/week, 50 min duration, 50–85% 1-RM, 5–8 OMNI-RES levels/strong intensity; 12 weeks, three times/week, 50 min duration/strong, 5–8 OMNI-Resistance Exercise Scale or RES levels intensity) in older women increased IGF-1 levels and the IGF-1/insulin-like growth factor-binding protein (IGFBP)3 ratio. Land resistance training had greater effect on IGF-1 levels [72].

In rats with cerebral infarction, strength training allowed a faster induction of long-term potentiation in the hippocampal Cornu Ammonis (CA)3 region and improved NMDA receptor channel function (increased open conductance, rate, and time) compared with non-trained infarcted controls. These exercise adaptations indicate that post-stroke exercise facilitated the recovery of excitatory synaptic transmission and enhanced hippocampal synaptic plasticity [73].

Progressive strength training in rats (4 weeks, 50–100% of maximal carrying capacity, frequency not indicated) increases neurogenesis, as suggested based on Ki-67-positive cells, but without changes in the BDNF level in the hippocampus [74]. In rats, strength training (8 weeks, 3–4 days/week, 40–50 min duration, 50–100% of intensity) and aerobic training (8 weeks, 3–4 days/week, 50 min duration, 13–17 m/min of intensity) exert similar effects on BDNF levels and neuroplasticity [75]. The levels of pNMDA, which plays an important role in learning, memory, and strength of excitatory synapses [76], are equally enhanced after training for both exercise protocols compared to the untrained animals [75].

### 3.4. Mind–Body Exercise

Mind–body exercises, such as yoga, tai-chi and dance, integrate physical movement with cognitive concentration and profound breathing. These behaviors are associated with decreased stress and improved psychological well-being [77]. In studies in MS women, a dance program of 18 weeks [78] and an 8-week pilates program [79] enhanced BDNF.

As previously reported, “dance is a multifaceted activity that includes physical exercise and cognitive, social, and artistic components, which are linked to visual–spatial, cognitive, and executive functions in individuals”, and its appeal seems ever increasing [80]. Interestingly, the volume in the left precentral gyrus, essential for the control of voluntary motor functions [78] increases after 6 months in dancers more than the subjects of sport groups, and, after another 12 months of training, an additional volume increase is observed in the right parahippocampal gyrus of the dancers, which is important for working memory [78].

These increases are not due to cardiovascular fitness levels, which remained constant in both groups, but are likely based on the complex and ever-changing movement patterns that the dancers had to perform, and due to the BDNF increase [78]. Abdullah et al. investigated the effects of a task balance training program in older adults (4 weeks, three times/week, 30 min per session, 30–40% of HRmax), showing a significant increase in GDNF levels, which is involved in neural plasticity, particularly in brain trauma and cognitive and memory impairment. Elevated GDNF levels can increase nerve cell survival in the nigrostriatal and other areas of the cerebral cortex [81]. This suggests the role of balance training in neurotrophic support [81].

**Figure 1:**
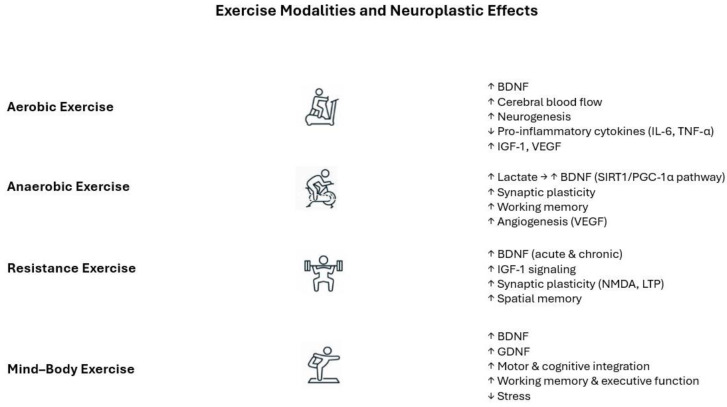
Exercise modalities and neuroplastic effects. Aerobic, anaerobic, resistance, and mind–body exercises modulate brain plasticity by distinct but partially overlapping molecular and functional mechanisms, including neurotrophin release, angiogenesis, synaptic plasticity, and cognitive enhancement. Abbreviations: BDNF, brain-derived neurotrophic factor; GDNF, glial cell-derived neurotrophic factor; IGF-1, insulin-like growth factor-1; VEGF, vascular endothelial growth factor; NMDA, N-methyl-D-aspartate receptor; LTP, long-term potentiation; IL-6, interleukin-6; and TNFα, tumor necrosis factor-alpha. Arrows indicate the modulatory effects, either positive or negative, displayed by distinct exercise regimens on neuroplastic and molecular pathways.

Figure 1 summarizes the neuroplastic impact of the main exercise categories.

It is mandatory to underline that most of data comes from experimental studies, especially in rodents, which are characterized by important differences in comparison with humans. As an example, in animals, the exercise-sustained, region-specific upregulation of central BDNF directly links to neurogenesis and synaptic remodeling, whereas human studies rely on peripheral BDNF measurements, which show transient responses and may reflect muscle sources, not neuronal synthesis [51].

Concerning IGF-I, studies on rats clearly demonstrate that exercise increases its uptake by brain, eliciting neuronal accumulation and, in turn, increasing sensitivity to afferent stimulation, spontaneous firing rate, and hippocampus activity [82]. Differently, investigations in humans report that peripheral IGF-I can rise, decrease, or be stable after exercise, independently from cognition improvement [83]. AD patients respond to exercise with IGF-I increase, likely for compensatory induced effects [84].

Similarly, VEGF-mediated neuroplastic effects or NF-kB modulation are directly demonstrated in animal brain areas like the hippocampus, while in humans they are largely inferred from peripheral measures and are not always detectable [85,86,87,88,89,90].

It should be noted that animal models resembling neurodegenerative diseases rely on induced neurodegeneration and often mirror a kind of “acute” stage rather than chronic condition as it is, indeed, in humans. Furthermore, animal exercise paradigms rely on a high degree of experimental control in contrast with the variability of human exercise interventions due to several factors, i.e., home-based or in-presence exercise, and RPE is used as an intensity parameter, interfering with animal/human comparison and transition.

Thus far, translational inference from rodents to humans is therefore biologically indirect. To date, electroencephalography (EEG) recordings can detect the exercise-induced modulation of human brain activity and reveal neuroplasticity response in the human cortex, but this effect is not recordable in some neurodegenerative disease, as in AD. Indeed, in AD mouse models the brain activity monitored with EEG does not change after exercise, differing from healthy control animals, suggesting that the presence/absence of exercise-specific changes in brain activity could be a tool to identify cognitive alterations [91].

## 4. Exercise as Hormetic Intervention to Sustain Synaptic Plasticity: Biomolecular Bases

Based on the principle that repeated exposure to low levels of environmental stimuli allows improvements in cellular and organ fitness and improves survival, exercise represents an excellent example of the hormetic method used to lower the burden of neurological disorders. Mild-to-moderate regular exercise is widely acknowledged to engage signal transduction pathways and gene expression programs that promote human health, while extreme intensities/durations without adequate rest resemble the symptoms of inflammation and elicit negative outcomes [92]. The emerging interest is in interventions able to interfere with these processes, in order to limit cell damage and neurodegeneration.

Exercise has been demonstrated to act as a pleiotropic intervention, engaging multiple molecular pathways that restore synaptic plasticity and attenuate inflammatory cascades. At the synaptic level, the impact of exercise on neurotrophic factor expression has been demonstrated, with the upregulation of BDNF, insulin-like growth factor-1 (IGF-1), and vascular endothelial growth factor (VEGF) being particularly notable. These factors have been observed to promote dendritic spine remodeling, long-term potentiation, and angiogenesis, thereby enhancing connectivity and metabolic support [93,94]. Peripheral exerkines, including myokines such as irisin (FNDC5) and cathepsin B, have been observed to cross BBB and stimulate BDNF expression, thereby establishing a link between muscle activity and central neuroplasticity [95,96,97,98].

Exercise activates the 5’ adenosine monophosphate-activated protein kinase/sirtuin1/peroxisome proliferator-activated receptor gamma coactivator 1-alpha (AMPK/SIRT1/PGC-1α) axis, improving mitochondrial biogenesis and oxidative capacity, which are critical for synaptic maintenance under stress conditions [99,100]. Importantly, exercise exerts potent anti-inflammatory effects by inhibiting NF-kB signaling, reducing pro-inflammatory cytokines, like tumor necrosis factor (TNF)α, and interleukin (IL)-1β), and promoting microglial phenotypes via triggering receptor expressed on myeloid cells (TREM)2 activation, thereby mitigating neuroimmune dysregulation [99,101,102]. These integrated mechanisms highlight exercise as a systemic modulator of neuroplasticity and neuroimmune homeostasis, thus offering therapeutic potential for neurodegenerative disorders. Overall, exercise increases neurotrophic factor production, modulates neuroinflammation, and strengthens synaptic plasticity in different neurodegenerative conditions [35,103], as reported in the following examples.

### 4.1. Neuroimmune Homeostasis: Exercise-Based Interventions for Neurodegenerative Diseases

#### 4.1.1. Amyotrophic Lateral Sclerosis

ALS is a neurodegenerative disease in adults characterized by the selective degeneration of both the upper and lower motor neurons, resulting in progressive muscle wasting and weakness [104]. Clinical motor features are highly heterogeneous among affected individuals. The impact of exercise training in ALS remains debated [105]. However, current evidence indicates that moderate, carefully structured exercise can enhance muscle strength, functional capacity, and quality of life in patients with ALS without accelerating disease progression [105].

ALS models have been shown to exhibit early synaptic dysfunction and neuroinflammation, which occurs prior to motoneuron death [104,106]. A study by Laszlo and colleagues [107] in a human post-mortem cortex demonstrates a decrease in the expression of postsynaptic proteins and BDNF signaling, suggesting an impairment in glutamatergic transmission. In superoxide dismutase SOD1-G93A mice, synaptic alterations include reduced dendritic spine density and excitatory/inhibitory imbalance, coupled with NF-kB activation and microglial pro-inflammatory states [108,109]. It is also interesting to note the differences between distinct effects exerted by different types of exercise. In the study conducted by Just-Borràs et al. [110], the SOD1-G93A mice were subjected to training that involved either running or swimming. Despite the significant disparities in the molecular mechanisms involved, the ultimate result is nevertheless favorable, as it leads to a reduction in the substantial alterations observed in the BDNF/TrkB pathway. Exercise interventions in ALS mice activate the AMPK/SIRT1/PGC-1α axis, enhancing mitochondrial biogenesis and oxidative capacity while simultaneously upregulating BDNF and IGF-1 levels in the spinal cord and muscle [99,105].

In patients with ALS, several exercise training protocols have been investigated to assess their potential therapeutic benefits [105], but very few were also evaluated in clinical trials with disclosed results. The interpretation of the obtained results is often challenged by the considerable intrinsic heterogeneity within the ALS population [105].

#### 4.1.2. Alzheimer’s Disease

Synaptic loss is a major contributor to cognitive decline in AD [111,112,113]. Human AD brain tissue exhibits reduced expression of neurotrophins BDNF and TrkB, impairing LTP and dendritic spine maintenance [93,114,115]. In APP/Presenilin PS1 and 5xfamilial Alzheimer’s Disease (FAD) mouse models, treadmill exercise restores synaptic plasticity through the upregulation of BDNF and the activation of AMPK, while reducing amyloid-induced NF-kB signaling and microglial activation [116,117,118]. Lactate, elevated during exercise, acts as a neuromodulator enhancing synaptic protein expression and cognitive performance in AD mice; blocking lactate transport abolishes these benefits [62,119].

It has been demonstrated that the implementation of physical exercise, regardless of its type or intensity, confers a positive effect on the progression of AD. This observation is supported by the evidence that physical exercise has been associated with an increased expression of Postsynaptic Density Protein 95 (PSD95), a key factor in establishing the precise structural architecture for rapid synaptic transmission and contributing to synaptic plasticity [120,121,122,123,124]. Furthermore, exercise increases the number of synapses and the dendritic spine density, and prevents the impairment of synaptic transmission via LTP [125,126,127,128].

Exercise also attenuates NLRP3 inflammasome activation and promotes hippocampal neurogenesis [129]. Clinical trials employing different exercise protocols show that structured, multimodal exercise programs can lead to meaningful improvements in global cognition and quality of life in people with Alzheimer’s disease.

#### 4.1.3. Parkinson’s Disease

PD pathology involves dopaminergic degeneration and cortico-striatal synaptic dysfunction [94,130,131]. In individuals with PD, aerobic exercise has been shown to elevate neurotrophic factors such as BDNF and GDNF, which are associated with dopaminergic neuron survival and improved synaptic connectivity [132,133]. Exercise reduces neuroinflammation by downregulating NF-kB and promotes mitochondrial function via AMPK/PGC-1α activation, which also reduces α-synuclein aggregation [99,134]. Myokines such as irisin improve mitochondrial health and mitigate oxidative stress in PD models, highlighting muscle–brain crosstalk as a pivotal mechanism [135]. Human studies using transcranial magnetic stimulation (TMS) demonstrate that exercise enhances corticomotor excitability and plasticity, although responses vary by disease stage [136,137].

Overall, clinical trials show that structured exercise serves as an effective intervention for improving sleep quality and enhancing physical function in individuals with Parkinson’s disease. These findings highlight the importance of integrating regular exercise into comprehensive management strategies for PD.

#### 4.1.4. Huntington’s Disease

HD models manifest severe synaptic dysfunction and excitotoxicity [138,139,140]. In R6/2 mice, exercise enhances BDNF and IGF-1 levels, promoting synaptic integrity and mitigating striatal degeneration [141,142]. Human pilot studies indicate that a single session of moderate-intensity aerobic exercise improves motor learning in premanifest HD patients, though neuroplasticity responses may be attenuated compared to controls [143,144].

#### 4.1.5. Multiple Sclerosis

MS is a multifaceted autoimmune disease of the central nervous system that causes inflammatory demyelination and axonal damage, leading to synaptic dysfunction and neuroinflammation, thus impairing motor pathways and producing muscle weakness, most notably in the respiratory muscles [145,146]. Individuals with MS may experience sensory disturbances, motor impairments, sleep abnormalities, fatigue, and cognitive or psychological symptoms [145,146]. Respiratory function is also compromised, further limiting walking capacity and diminishing one’s overall quality of life [145,146]. Worsening motor performance and chronic fatigue over the disease course limit patients’ ability to be physically active and accelerate the deterioration of pulmonary function. These observations highlight the relevance of respiratory muscle training. Several studies agree on the beneficial effects of breathing training in patients with MS, which not only improves respiratory function but also enhances thoracic stability and trunk control by strengthening the diaphragm and accessory respiratory muscles [147].

Importantly, MS is defined by a disrupted inflammatory equilibrium that favors proinflammatory processes; this suggests that exercise training could play a role in modulating this immune dysfunction. A combined exercise program consisting of 24 sessions over 8 weeks, each including a warm-up, stretching, aerobic activity, and resistance–endurance training showed decline in disability scores while significantly improving muscle strength and balance [148]. Notably, the levels of IL-17 and IFN-γ in the plasma and PBMCs of trained MS patients were reduced, indicating that combined exercise training may exert beneficial anti-inflammatory effects, particularly through lowering IL-17 production [148].

As mentioned above, neurotrophic factors play key roles in neuroprotection, neuroplasticity, and overall neuronal maintenance. Two studies, an 8-week aerobic exercise (30 min of leg cycling at 60% VO2peak, 2 days per week) [149] and a single-group pre–post (30 min of leg cycle ergometry at 60% VO2peak, 3 days per week) [150], reported no changes in circulating BDNF, NGF, or IGF-1 levels after aerobic exercise training. Thus, exercise does not alter systemic neurotrophic factor levels in people with MS. However, experimental autoimmune encephalomyelitis (EAE), the predominant animal model of MS, shows that moderate treadmill exercise reduces inflammatory infiltrates, downregulates pro-inflammatory cytokines, and promotes synaptic integrity in the spinal cord and hippocampus [151]. Notably, exercise modulates the kynurenine pathway, reducing neurotoxic metabolites (quinolinic acid) and increasing neuroprotective kynurenic acid, thereby decreasing excitotoxicity and oxidative stress [152,153]. In patients with MS, TMS studies reveal that preventive exercise enhances LTP-like plasticity and corticospinal connectivity, while bilateral upper limb training improves motor conduction and synaptic responsiveness [154,155].

Overall, exercise training in people with MS consistently improves muscle strength, aerobic fitness, and walking ability, with supportive evidence for benefits in fatigue, balance, gait, and quality of life [156]. However, there is no strong evidence that exercise also affects inflammation, neurodegeneration, cognition, or daily functioning. Research is limited, highlighting the need for further studies to fully understand the potential cellular and functional benefits of structured physical activity in MS.

#### 4.1.6. Spinal Muscular Atrophy

SMA is an autosomal recessive disorder affecting lower motor neurons, marked by the progressive weakness and wasting of proximal muscles. Depending on the severity, patients may also experience respiratory problems, sleep disturbances, psychological symptoms, impaired postural control, and skeletal deformities. SMA affects approximately 1 in 10,000–20,000 newborns worldwide. As a major genetic cause of infant mortality, SMA arises from biallelic loss of *SMN1* gene, most commonly homozygous exon 7 deletion or heterozygous mutations, leading to SMN protein deficiency, the degeneration of α-motor neurons in the anterior horn of the spinal cord, and consequent muscle weakness and wasting [157]. As mentioned, SMA presents with a wide spectrum of clinical features and is commonly classified into four distinct subtypes based on the age at symptom emergence and the highest motor abilities achieved [157].

Nusinersen, risdiplam, and onasemnogene abeparvovec significantly extend survival and improve functional outcomes in individuals with SMA. Nevertheless, patients often continue to experience a broad range of symptoms, including bradykinesia and postural instability [158]. Consequently, comprehensive multidisciplinary management remains essential, with rehabilitative interventions including tailored exercise regimens. In type 2 and type 3 SMA-like mouse models, long-term exercise protocols (high-intensity swimming vs. low-intensity running) have been shown to significantly improve motor neuron survival, maintain neuromuscular junctions, and reduce muscle fatigability, independently from SMN expression [159,160]. Mechanistically, exercise has been shown to enhance N-Methyl-D-aspartic acid (NMDA) receptor activity in spinal motor neurons, particularly the NR2A subunit, thereby promoting motor unit maturation and synaptic excitability [161]. These adaptations have been shown to correlate with improved motor behavior and metabolic resilience, underscoring the notion that exercise serves as a significant modulator of synaptic and neuromuscular plasticity in SMA.

The treatment of patients with SMA comprehends a broad supportive framework combining rehabilitative therapy with orthopedic management, respiratory care, and nutritional support. Studies have examined interventions ranging from hydrotherapy and aerobic conditioning to strengthening exercises, stretching routines, and balance or gait training, suggesting some potential improvements in posture and movement [162]. Nevertheless, the current body of evidence remains far from definitive, and studies evaluating rehabilitation outcomes in SMA often yield divergent and sometimes contradictory conclusions [163]. A randomized, controlled trial of aerobic and strengthening exercise in 14 ambulatory SMA patients ranging in age from 8 to 50 years showed improved VO2max in all participants in 6 months, supporting the conclusion that regular exercise is well tolerated in ambulant SMA, with no detrimental impact on motor ability or fatigue [164]. Notably, participants demonstrated lowered oxidative capacity [164]. These physiological abnormalities may be linked to mitochondrial loss in SMA, underscoring the need for additional research.

Despite the predominance of in vitro and animal studies, which may not fully capture human pathophysiology, several clinical trials have begun to investigate the effects of exercise-based interventions in neurodegenerative diseases. However, although numerous clinical trials have been registered to investigate treatments for neurodegenerative diseases, only a limited number have disclosed and published their findings, summarized in Table 1.

Besides disease-dependent heterogeneity, due to disease type, stage/severity, pharmacological treatment, and co-morbidity presence, other factors likely contribute to the discrepancies found in the results from clinical trials, i.e., the lack of design homogeneity, the small sample size (due to enrollment difficulties), and the difference in outcomes (often only focused on cognitive skills). Importantly, the differences found in exercise regimens, not only limited to exercise type, but including the duration, frequency or intensity, and the choice of professional instruments for anthropometric measurements, contribute to the discrepancies observed in trials.

In this scenario, the major challenge is to find/set dedicated facilities and counteract kinesiophobia. In fact, dedicated clinical research centers capable of integrating exercise interventions with systematic biomolecular monitoring (i.e., BDNF, IGF-1, VEGF, and inflammatory markers) should be strongly encouraged. These structures, indeed, would enable the standardization of exercise protocols based on molecular responses, facilitating a more precise and biologically grounded approach to enhancing synaptic plasticity. To the writers, this seems a critical point to begin translating the personalized treatments into clearer evidence-based guidance. In addition, it is likely that patients involved in exercise-based common sessions, within dedicated spaces facilitating socialization, are more prone to overcome their fear of moving.

Anyhow, the observation that different exercise regimens display distinct effects gives the possibility to design adapted and personalized treatments with tailored exercise.

## 5. Conclusions

Thus far, it is undeniable that exercise positively shapes several biomolecular signaling underlying neurodegenerative diseases; therefore, it potentially represents a promising tool to contain neurological illness burden.

It is mandatory to underline that, despite the well-established role of exercise in sustaining synaptic plasticity at the molecular level, a relevant translational gap persists between animal models and human studies. In animal settings, exercise-induced adaptations are supported by direct evidence of central neurotrophins’ regulation, neurogenesis, and synaptic remodeling. In humans, these mechanisms are primarily inferred from peripheral biomarkers, which may not accurately reflect brain-specific processes. This gap is further amplified by the heterogeneity of exercise interventions in human studies, which are often home-based, unsupervised or characterized by broad intensity prescriptions (i.e., rating of perceived exertion), limiting precise dose–response analyses. Such variability contrasts with the highly controlled exercise paradigms typically adopted in animal models and may contribute to the inconsistent molecular findings observed in clinical research and trials.

Finally, albeit the mechanisms that regulate synaptic plasticity are common, differences emerge when a specific type of disease develops (due to multifactorial causes), involving and developing a specific microenvironment—i.e., different protein accumulation and, consequently, different aberrant signaling cascades. These differences in the biomechanisms involved in cascade determine different responses, even within the same pathology, depending on disease duration, age, and the general state of the subject.

Altogether, considering these issues, the need for fine-tuned personalized protocols is undeniable. Therefore, more structured and standardized exercise protocols in humans, designed and supervised by kinesiologists, professionals with specific expertise in exercise prescription to ensure appropriate modulation of training variables including intensity, volume, frequency, and progression, are mandatory. Furthermore, better integrated communication among professionals who take care of the patients (i.e., neurologists, neuropsychologists, physiatrists, physiotherapists, and kinesiologists) should be strongly encouraged.

It is worth noting that there are not yet disease-modifying treatments for neurodegenerative pathologies, which are expanding also in parallel with the increasing number of elderly subjects. Hence, these diseases, which currently pose a clinically unmet need, are likely to further impact medical and social areas, with related public expending increase. There is an urgent need, undeniably, to provide social, dedicated centers to help patients and their families/caregivers. In this scenario, exercise emerges as a promising intervention capable of counteracting the decline of synaptic plasticity, the capacity to adapt to changes progressively lost in neurodegeneration. The ability to adapt, as part of hormetic response, offers a framework to intervene with different protocols of exercise in the therapeutic approach.

Even though neurodegenerative diseases represent, unquestionably, a critical issue, socially, clinically, and economically, some related aspects are still quite neglected. Moreover, the literature covers as separate topics the biomolecular mechanisms underlying synaptic plasticity decline/loss as targets of exercise and reports do not delve into the effectiveness of different exercise regimens. Also, the general features of the different exercise regimens give only some indications on the best possible associated exercise–target(s) within a certain disease type, but they are not sufficient to design a personalized protocol. Herein, we try to focus on the need for an overall view within an integrated vision to approach neurodegenerative diseases.

Further basic and clinical studies are mandatory in this field aimed to clarify scientific aspects and, not less important, to capture the attention of researchers and clinicians on integrated treatments including exercise. So far, the current challenge is not only how to shape and personalize exercise according to patients’ needs, but to introduce it within the therapeutic approach in neurodegenerative diseases.

## Figures and Tables

**Table 1:** Clinical trials with published results employing exercise-based interventions for neurodegenerative diseases.

Clinical Trial ID	Disease	Outcome	Reference
NCT02727478	Parkinson’s Disease	Balance and gait improvement.	[165,166]
NCT00784563	Parkinson’s Disease	Improvement of aerobic fitness, motor function, fatigue, and mood.	[167]
NCT05756075	Parkinson’s Disease	Ongoing analysis.	[168]
NCT02384993	Alzheimer’s disease	Improvement in executive function correlated with increased VO2peak.	[169]
NCT01504958	Alzheimer’s disease	Cognitive improvement.	[170]
NCT00591344	Parkinson’s Disease	Improvement of attention and working memory.	[171]
NCT01128361	Alzheimer’s disease	Improved memory performance and reduced hippocampal atrophy.	[172]
NCT03213873	Parkinson’s Disease	Improvement of loudness, articulation, and voice quality.	[173]
NCT01768832	Parkinson’s Disease	Treadmill improved forward walking and backward walking improved with treadmill and stretching.	[174]
NCT03555695/ NCT03882879	Parkinson’s Disease	Quality of life and wellbeing improved significantly.	[175]
NCT01490840	Multiple Sclerosis	Reduced fatigue in subgroups of PwMS.	[176]
NCT03658668	Multiple Sclerosis	A single session of tDCS is not sufficient to improve walking and functional mobility.	[177]
NCT03256851	Multiple Sclerosis	Telephone-delivered exercise intervention that targets fatigue is feasible and acceptable	[178]
NCT03801473.	Multiple Sclerosis	robot-assisted gait training (RAGT) and conventional gait training are effective. RAGT has superior effects in terms of fatigue, depression, and anxiety.	[179]

## Data Availability

No new data was created or analyzed in this study. Data sharing is not applicable to this article.

## Abbreviations

The following abbreviations are used in this manuscript:

Aβ	Amyloid Beta
AD	Alzheimer’s Disease
ALS	Amyotrophic Lateral Sclerosis
AMPK	Adenosine Monophosphate-activated Protein Kinase
ATP	Adenosine Triphosphate
BBB	Blood–Brain Barrier
BDNF	Brain-derived Neurotrophic Factor
CAMP	Cyclic Adenosine Monophosphate
CREB	cAMP-Response Element Binding Protein
CNS	Central Nervous System
DAMPs	Damage-associated Molecular Patterns
EAE	Experimental Autoimmune Encephalomyelitis
FAD	Flavin Adenine Dinucleotide
GDNF	Glial Cell-derived Neurotrophic Factor
GLPD1	Glucagon-Like Peptide D1
HD	Huntington’s Disease
HIIT	High Intensity Interval Training
HRR	Heart Rate Reserve
IGF-1	Insulin-like Growth Factor-1;
IL-1β	Interleukin 1 beta
IL-6	Interleukin-6
LTD	Long-Term Depression
LTD	Long-Term Potentiation
MAPK	Mitogen-Activated Protein Kinase
MS	Multiple Sclerosis
NF-kB	Nuclear Factor kappa-light-chain-enhancer of Activated B Cells
NGF	Nerve Growth Factor
NMDA	N-Methyl-D-aspartic acid
Nrf2	Nuclear Factor Erythroid 2–Related Factor 2
NLRP3	Nucleotide-binding Oligomerization Domain-Like Receptor [NLR] Family Pyrin Domain-Containing 3
NT	Neurotrophine
PAMPs	Pathogen-Associated Molecular Patterns
PD	Parkinson’s Disease
Pi3k	Phosphatidylinositol 3-kinase
PSD95	Postsynaptic Density Protein
qoL	Quality of Life
RAGT	Robot-Assisted Gait Training
RPE	Rate of Perceived Exertion
ROS	Reactive Oxygen Species
SIRT1	Sirtuin 1
SMA	Spinal Muscular Atrophy
tDCS	Transcranial direct current stimulation
TLR	Toll-like Receptor
TMS	Transcranial Magnetic Stimulation
TNFα	Tumor Necrosis Factor Alfa
TREM	Triggering Receptor Expressed on Myeloid Cells
VEGF	Vascular Endothelial Growth Factor
